# An open GIS based 3D simulation software to predict cooling tower drift diffusion

**DOI:** 10.1038/s41598-023-45293-y

**Published:** 2023-10-24

**Authors:** Xuan Wang, Minghua Lv, Shuhuan Liu, Jing Li, Junfang Zhang, Fanjun Meng

**Affiliations:** 1 School of Energy and Power Engineering, Xi’an Jiaotong University, Xi’an, 710049 China; 2 Department of Nuclear Environmental Science, China Institute for Radiation Protection, Taiyuan, 030006 China; 3 MSC CFD-Cradle BD China, MSC Software Corporation, Beijing, 100048 China

**Keywords:** Ecology, Computer science, Software

## Abstract

This paper developed XJCT-3D, a simulation software for cooling tower wet plume dispersion. By coupling it with the Open GIS component Dotspatial, we have achieved geospatial visual representation of the calculation results, which has solved the problems of low calculation efficiency and insufficient visual representation of the traditional CFD software in the calculation of cooling tower wet plume dispersion. In order to verify the validity of the XJCT-3D software simulation results, we have conducted tracer experimental data from the ChalkPoint power plant. XJCT-3D accurately models wet plume deposition during cooling tower operation. From the XJCT-3D calculation results, we have observed that the maximum value of the cooling tower thermal plume wet deposition occurs near 610 m with a maximum value of 6.9E−07 kg/m^2^ s. This finding suggests that the cooling tower emissions carry a significant load of particles or droplets that have settled on surfaces at this particular altitude. It provides insights into potential environmental and human health impacts and helps in identifying and assessing areas at relatively higher risk of deposition, such as nearby ecosystems, farmland, or urban areas. This information can contribute to the development of effective mitigation strategies and the implementation of appropriate measures to minimize the impact of cooling tower emissions.

## Introduction

During the operation of large natural draft cooling towers, a significant amount of water vapor is generated, forming wet plume that affects the surrounding landscape environment and impacts the local air humidity. If water vapor collects under the influence of the structure, it can also create a wide range of dark clouds, resulting in solar radiation loss and, in severe cases, reducing local crop yields^1,2^. For large natural draft cooling towers using seawater, the wet plume also contains a significant amount of salt particles. Depositing these particles in agricultural land can cause vegetation death, concentrate on building surfaces, and have a corrosive effect^3,4^. Therefore, conducting a computational analysis of wet plume dispersion in large naturally ventilated cooling towers is important to assess these environmental impacts.

With the ongoing development of numerical simulation theory, the utilization of 3D numerical methods to carry out cooling tower wet plume dispersion analysis, together with the implementation of GIS technology for the visual presentation of calculation results, has captured the attention of scholars in the field^5,6^. This paper focuses on constructing a quick 3D numerical simulation and analysis platform to enable prompt analysis and visualization of heat plume diffusion in cooling towers.

In this paper, the study of the diffusion process of the wet heat plume of the cooling tower includes 2 main aspects: (a) Analysis of the dynamic and thermal lifting process of the cooling tower heat plume; (b) Analysis of the diffusion trajectory of the visible heat plume of the cooling tower. Hanna^7^ pointed out that Briggs’ formula^8^ on the lift of a dry plume can be applied to calculate the lift of cooling tower water vapor, but the buoyancy flux should include the increased buoyancy due to the heat released by the latent heat of condensation of water vapor, and other parameters can be calculated by applying the virtual temperature of specific humidity, etc., although this approach has been questioned by other scientists. Weil^9^ gave an analytical expression for the saturated plume lift trajectory, the maximum lift height, and the downwind distance when the maximum height is reached in a saturated environment under steady conditions, which is of great practical importance. Meanwhile, Weil gives an example of calculation. He found that when the ambient temperature gradient was Г =  − dT/dz = 0 and Г = 0. 0055 K/m, the ratio of the maximum lifting height of the saturated plume to the maximum lifting height of the dry plume in the isothermal environment was 1.18 and 1.93, respectively, which means that the maximum lifting height of the wet plume increased by 18% and 93%, respectively, while the ratio of the maximum lifting The ratios of the maximum lifting distances were 1.21 and 2.56 respectively. Wigley’s numerical calculations show that the more stable the atmospheric boundary layer is, the greater the ratio of the lift height of cooling tower water vapor to the lift height of dry flue gas. Finally, Hanna gives a set of empirical formulas for calculating the height and length of visible water vapor in cooling towers assuming that the environmental variables do not change with height. For the study of the cooling tower heat plume diffusion trajectory, more famous is the ChalkPoint experiment in the 1980s^10^. The U.S. NRC has developed an analytical calculation model of cooling tower plume diffusion, known as SACTI^11^, based on experimental data. The model operates under the basic principle of the Gaussian linear plume and can utilize year-round meteorological data to assess the cooling towers environmental impact, providing values for annual average and seasonal variations. However, due to the model's failure to consider the impact of large naturally-ventilated cooling towers on the local flow field, the results of the calculations are often excessively conservative.

The application of CFD technology can effectively solve the influence of buildings on the diffusion of cooling tower heat plumes, Robert^12^ and others carried out the diffusion analysis of cooling tower heat plume based on Fluent software, and Guo^13^ used Star-CD software to carry out the heat plume lift analysis of cooling towers, but the traditional CFD software still has the following problems when carrying out the heat plume diffusion analysis of cooling towers:

- The computational process is more complex, and analysts need to spend a lot of time in the construction and meshing of the model.
- Simulation results cannot be expressed in spatial data, and designers cannot directly obtain the specific impact location of the cooling tower heat plume from the results.
- Calculation efficiency is too low. When evaluating cooling tower heat plume analysis, a large number of meteorological moments need to be considered, for example, 8760 h of the whole year are considered, which may take several months if the traditional 3D numerical simulation technique is used, which is unacceptable in practical engineering projects.

Thus, the present research gaps were identified, such as the lack of a comprehensive method for cooling tower drift diffusion prediction that incorporates environmental factors and multiple sources of drift emission. It was also observed that most existing methods do not consider the impact of complex terrain and building structures that can affect drift dispersion.

Given the above problems, this paper applies the Cradle CFD^14^ calculation engine and develops a three-dimensional numerical analysis platform (XJCT-3D) for cooling tower atmospheric dispersion, which realizes the rapid analysis and calculation of the calculation process, while coupling with Open GIS^15^ technology to realize the spatial display of the calculation results. This paper consists of the following sections: “Calculation equations” section introduces the basic architecture of the software and the computational methods. “Methods to develop the software” section introduces the software development process, including the construction of the cooling tower model, rapid delineation of the grid, real-time computational analysis, post-processing of the computational results, and GIS coupling analysis techniques. “Case study” section takes a nuclear power plant site in China as an example to illustrate the application of the software.

This research will be conducted utilizing Computational Fluid Dynamics (CFD) technology to analyze and simulate fluid flow phenomena in a controlled environment. Specific software, such as Cradle CFD, will be employed to create virtual models and simulate fluid behavior.

The research methodology will involve several steps. Firstly, a detailed literature review will be conducted to gather relevant information and establish a theoretical framework. This will ensure that the research is built upon existing knowledge and addresses any research gaps.

Next, the research will involve the creation of a virtual model, representing the physical system of interest. This will be done using specialized CAD software and will accurately capture the geometry, dimensions, and boundary conditions of the system.

After setting up the virtual model, the chosen CFD software will be utilized to perform numerical simulations. The software will solve the governing equations of fluid flow, such as the Navier–Stokes equations, in order to predict flow patterns, velocity distributions, pressure gradients, and other relevant parameters. The simulations will be run multiple times, taking into account different scenarios, design variations, or operating conditions to analyze their impact on the system.

Additionally, post-processing techniques will be applied to the simulation results to extract meaningful information and visualize the data. This may include the generation of contour plots, streamlines, or velocity vectors, allowing for a comprehensive understanding of the fluid flow behavior.

The simulations will then be validated by comparing the results with experimental data, if available. This validation step ensures the accuracy and reliability of the simulation model.

The primary objective of this study is to develop an open GIS based 3D simulation software to predict the cooling tower drift diffusion and assess its impact on the surrounding environment. To achieve this objective, the following had been done.

1. We have designed and implemented a simulation model that integrates various environmental and operational factors influencing the drift diffusion process.
2. We have focused on developing a software that is user-friendly and can be used by stakeholders including environmental regulators, industry operators, and researchers to predict the potential impact and optimize the design of cooling tower systems.

This research aims to delve deeper into the problem at hand and provide a comprehensive understanding of its underlying causes and potential solutions. By conducting in-depth analysis and employing advanced methodologies, this study will contribute to the existing body of knowledge in the field and shed light on previously unexplored aspects.

In terms of its benefits, this research has the potential to significantly impact both academia and the industry. Firstly, it will offer valuable insights to researchers and scholars, allowing them to expand their understanding of the subject matter and develop new theories or frameworks. Additionally, professionals in the relevant field can benefit from this research by gaining a better understanding of best practices and potential strategies to address the challenges associated with the problem. This knowledge can ultimately lead to improved decision-making, enhanced problem-solving capabilities, and more effective interventions.

Furthermore, the outcomes of this research will be useful for policymakers and stakeholders. By identifying key factors, barriers, or opportunities related to the issue at hand, policymakers can devise informed policies that promote positive change. Similarly, stakeholders such as businesses or organizations can leverage the findings to shape their strategies and initiatives, ultimately leading to improved outcomes and sustainable practices.

## Calculation equations

### The process of generating a cooling tower wet plume

Natural draft cooling towers rely on the air density difference between the inside and outside of the tower or the natural wind to form the air convection effect for ventilation. The main structure of the cooling tower includes an air inlet, filler spray area, and air outlet, at the outlet is generally equipped with a drip remover, the purpose of which is to eliminate the cooling tower wet heat plume contained in the large particle size moisture^16^. The warm water generated during the operation of the power plant is showered down through the filler spray area, forming a process similar to rainfall, in which the ambient air and “warm water rainfall” for heat exchange, the water temperature falling into the pool quickly, and then again into the cooling water system of the power plant, and so on. The heated air floats up the cooling tower body and is discharged from the cooling tower outlet, forming a wet heat plume.

The air inside the cooling tower is heated and lifted naturally, with a large amount of water vapor entrained in the air, forming a wet heat plume discharged from the top of the cooling tower. The particle size of the droplets in the wet plume is about 10–1000 μm, due to the small particle size and lightweight, can reach a certain distance under the natural wind blowing, and in a certain range to affect the solar radiation, forming a large area of shadow, known as the “shade screen”, thereby reducing the energy of solar radiation reaching the ground. For seawater cooling towers, salt deposits from the diffusion of the wet plume can also affect the surrounding vegetation and deposit on the building, which can also form corrosion to the building.

The process of generating a cooling tower fog plume involves several technical terms, which are briefly explained below:

1. *Air convection effect* This refers to the movement of air due to temperature differences. In a cooling tower, warm air rises and is replaced by cooler air, creating a convection effect that helps to enhance the cooling process.
2. *Filler spray area* The filler is a component inside the cooling tower that helps to increase the surface area for effective heat transfer. The filler spray area, therefore, refers to the specific section of the cooling tower where the water is distributed over the filler material to maximize contact with the air for efficient cooling.

**Figure 1:**
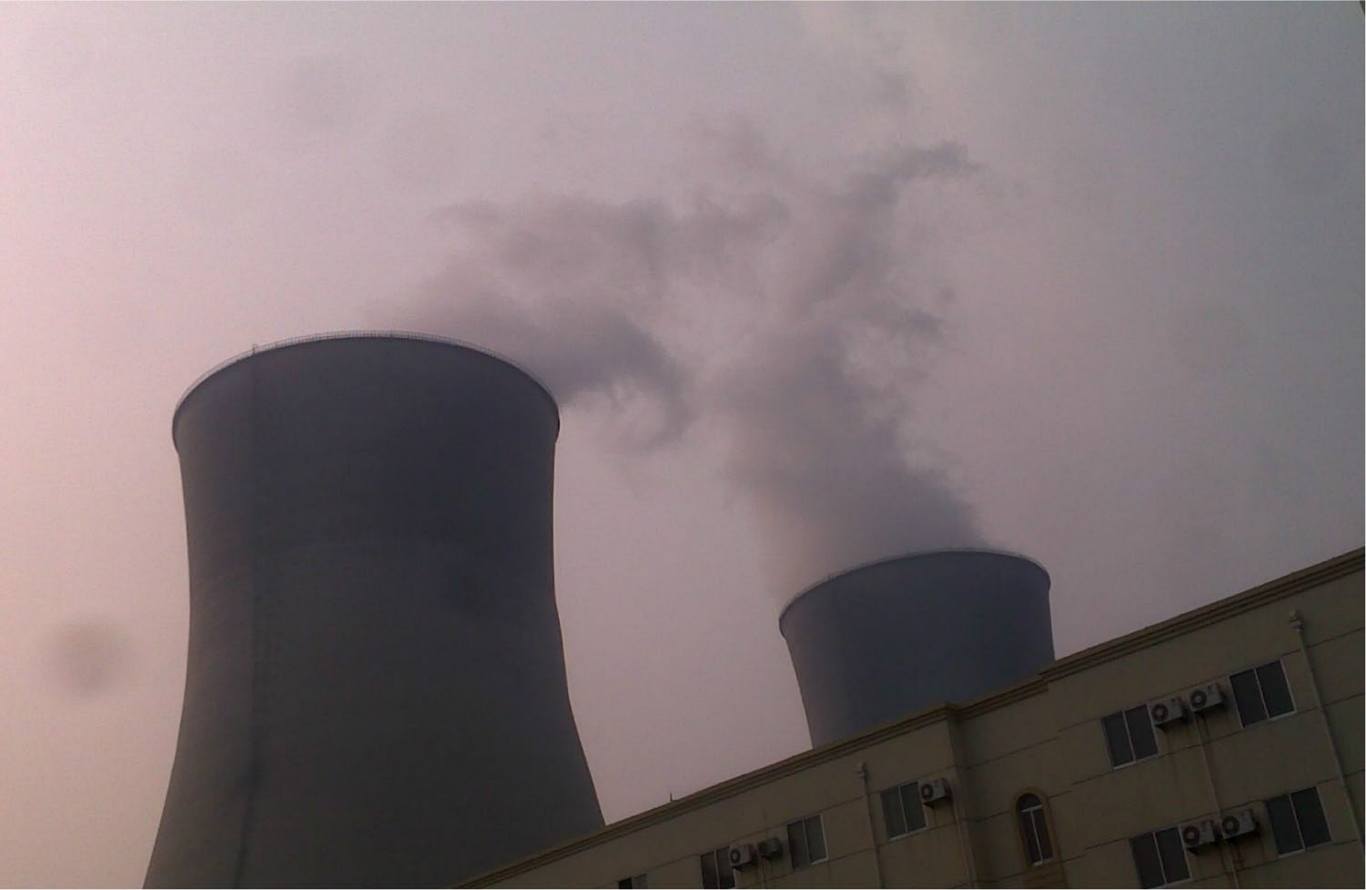
Moist wet plume discharged from cooling tower.

Cooling tower emissions of high temperature, high humidity water vapor mixed with ambient air, a series of heat exchanges, water vapor temperature decreases, when the water vapor is in a supersaturated state, and liquid water condensation, and finally high temperature, high humidity water vapor into droplets, that is, we usually speak of visible wet plume as shown in Fig. 1.

The lift height and length of the visible wet plume of a cooling tower can be calculated from Eqs. (1), (2), and (3) as follows:

1 $$ z = 9R_{0} \left[ {\left( {\frac{{q_{p0} + q_{l0} }}{{q_{s} - q_{e} }}} \right)^{\frac{1}{2}} - 1} \right]\quad ({\text{Windless}}), $$

2 $$ z = 3.6R_{0} \left( {\frac{{{\text{W}}_{{0}} }}{U}} \right)^{\frac{1}{2}} \left[ {\left( {\frac{{q_{p0} + q_{l0} }}{{q_{s} - q_{e} }}} \right)^{\frac{1}{2}} - 1} \right]\quad \left( {{\text{Windy}}} \right), $$

3 $$ l = 3.4\frac{{R_{0}^{{{3 \mathord{\left/ {\vphantom {3 2}} \right. \kern-0pt} 2}}} U^{{{3 \mathord{\left/ {\vphantom {3 4}} \right. \kern-0pt} 4}}} W^{{{3 \mathord{\left/ {\vphantom {3 4}} \right. \kern-0pt} 4}}} }}{{F_{0}^{{{1 \mathord{\left/ {\vphantom {1 3}} \right. \kern-0pt} 3}}} }}\left[ {\left( {\frac{{q_{p0} + q_{l0} }}{{q_{s} - q_{e} }}} \right)^{\frac{1}{2}} - 1} \right]^{{{3 \mathord{\left/ {\vphantom {3 2}} \right. \kern-0pt} 2}}} \quad \left( {{\text{Windy}}} \right), $$

where *z*—the lifting length of the visible wet plume in meters (m); *l*—length of the visible wet plume in meters (m); *q*_*p*0_—specific humidity of the cooling tower outlet wet plume in grams per gram (g/g); *q*_*l*0_—initial specific humidity of the cooling tower outlet wet plume liquid water in grams per gram (g/g); *q*_*s*_—ambient atmospheric saturation specific humidity in grams per gram (g/g); *q*_*e*_—ambient atmospheric specific humidity in grams per gram (g/g); *W*_0_—initial velocity of the wet plume at the exit of the cooling tower in meters per second (m/s); *F*_0_—the initial heat flux of the wet plume at the exit of the cooling tower, in meters to the fourth power per cubic second (m^4^/s^3^).

For a typical cooling tower, the cooling tower outlet water vapor discharge temperature is 30.33 °C, while the cold air temperature enters from the bottom of the cooling tower is only 16.7 °C.

### Basic control equations^17^

#### Mass conservation equation

The mass conservation equation, also known as the continuity equation, has the following basic expression:

4 $$ \frac{{\partial \left( {\rho v_{x} } \right)}}{\partial x} + \frac{{\partial \left( {\rho v_{y} } \right)}}{\partial y} + \frac{{\partial \left( {\rho v_{z} } \right)}}{\partial z} + \frac{\partial \left( \rho \right)}{{\partial t}} = 0, $$

where *ρ* represents the density of the fluid and is a constant; *v*_*x*_, *v*_*y*_, and *v*_*z*_ represent the velocity vector components of the fluid in the *x*, *y*, and *z* directions, respectively; *t* represents the time.

In a continuous incompressible fluid, the density of the fluid is treated as a constant.

5 $$ \nabla \cdot \left( {\vec{v}} \right) = 0 \cdot \left( {div\left( {\vec{v}} \right) = 0} \right). $$

#### Momentum conservation equation

The momentum conservation equation, also known as the equation of motion or Navier–Stokes (N–S) equation, has the following basic equation;

6 $$ \rho \left( {\frac{{\partial v_{x} }}{\partial t} + v_{x} \frac{{\partial v_{x} }}{\partial x} + v_{y} \frac{{\partial v_{y} }}{\partial y} + v_{z} \frac{{\partial v_{z} }}{\partial z}} \right) = f_{x} \rho \left( {\frac{{\partial \tau_{xx} }}{\partial x} + \frac{{\partial \tau_{yx} }}{\partial y} + \frac{{\partial \tau_{zx} }}{\partial z}} \right), $$

7 $$ \rho \left( {\frac{{\partial v_{y} }}{\partial t} + v_{x} \frac{\partial y}{{\partial x}} + v_{y} \frac{{\partial v_{y} }}{\partial y} + v_{z} \frac{{\partial v_{z} }}{\partial z}} \right) = f_{y} \rho \left( {\frac{{\partial \tau_{xy} }}{\partial x} + \frac{{\partial \tau_{yy} }}{\partial y} + \frac{{\partial \tau_{zy} }}{\partial z}} \right), $$

8 $$ \rho \left( {\frac{{\partial v_{z} }}{\partial t} + v_{x} \frac{\partial z}{{\partial x}} + v_{y} \frac{{\partial v_{z} }}{\partial y} + v_{z} \frac{{\partial v_{z} }}{\partial z}} \right) = f_{z} \rho \left( {\frac{{\partial \tau_{xz} }}{\partial x} + \frac{{\partial \tau_{yz} }}{\partial y} + \frac{{\partial \tau_{zz} }}{\partial z}} \right), $$

where *τ*_*x*_, *τ*_*y*_ and *τ*_*z*_ represent the molecular viscous stress components of the fluid in the* x*, *y*, and *z* directions, respectively.

By introducing Newton’s law of shear into the above equation and eliminating the stress term from the set of equations, the incompressible N-S equation with constant viscosity can be derived as

9 $$ \frac{{D\vec{v}}}{Dt} = \vec{f} - \frac{1}{\rho }\nabla p + v\nabla^{2} \vec{v}. $$

#### Energy conservation equation

Energy conservation equation is calculated as follows:

10 $$ \frac{DT}{{Dt}} = \frac{\lambda }{{\rho c_{p} }}\nabla \left( {gradT} \right) + \frac{{S_{T} }}{\rho }, $$

where *λ* represents the fluid thermal conductivity term; *S*_*T*_ represents the heat source correction term of the fluid; *ρ* represents the density of the fluid and is a constant.

#### Component mass conservation equation

In addition, to calculate the concentration of pollutants in the control volume, it is necessary to introduce the concept of components based on the above equation by adding the component mass equation, which is calculated as follows:

11 $$ \frac{{\partial \left( {\rho c_{S} } \right)}}{\partial t} + div\left( {\rho \mathop{\longrightarrow}\limits^{u}c_{s} } \right) = div\left( {D_{S} grad\left( {\rho c_{S} } \right)} \right) + S_{S} , $$

where *c*_*S*_ represents the volume concentration of component *S*; *ρc*_*S*_ represents the mass concentration of component *S*; *D*_*S*_ represents the dispersion coefficient of component *S*; *S*_*S*_ represents the mass production rate of the component, which is the release rate by atmospheric dispersion.

In the present model, it is assumed that the gas-phase in the cooling tower operates under ideal gas behavior. The droplet size distribution is assumed to be constant throughout the cooling tower, and the droplets are considered spherical and non-interacting. Additionally, it is assumed that the velocity and temperature fields are homogeneous and the heat transfer between the droplets and the surrounding gas is governed by convective heat transfer only.

## Methods to develop the software

**Figure 2:**
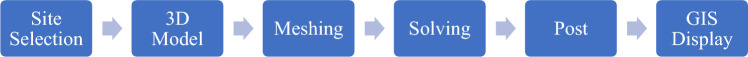
Flow chart of the research methodology.

The research methodology of this paper is shown in Fig. 2.

### Software architecture

**Figure 3:**
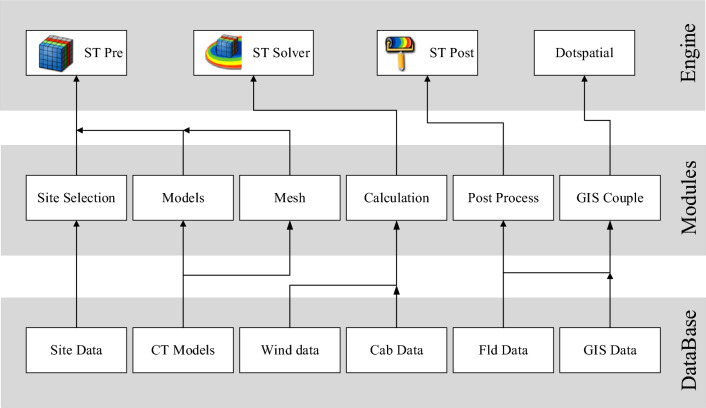
XJCT-3D software architecture.

The XJCT-3D software consists of three groups calculation engine, function module, and database, as shown in Fig. 3. The calculation engine is developed and completed based on Cradle CFD, the functional modules include plant site selection, 3D modeling, meshing, calculation, result post-processing, and GIS integration, the database includes plant site data, cooling tower 3D model data, wind boundary data, Cab project file data, Fld result file data, and GIS data.

#### Software component: 3D modeling module

- *Functional description* The 3D modeling module is a software component that allows users to create and manipulate three-dimensional models. It provides various tools and techniques to design, modify, and visualize objects in a virtual three-dimensional space.
- *3D modeling module* This module enables users to generate and manipulate virtual objects in a three-dimensional environment. It typically includes features like geometric modeling, texture mapping, lighting, and rendering to create realistic and visually appealing 3D models.

#### Term: wind boundary data

- *Definition* Wind boundary data refers to specific information regarding the boundary conditions of wind flow, such as velocity, direction, and turbulence, within a given computational domain. It is an important input parameter for simulations or analyses related to airflow, aerodynamics, or wind-related phenomena.

#### Term: cab project file data

- *Definition* The Cab project file data refers to the structured information stored in a file format that is specific to the Cab project. A Cab project file typically contains data related to the configuration, settings, inputs, and outputs of a particular project. It serves as a repository of project-specific information that can be loaded and accessed by the software for project management and analysis purposes.

**Figure 4:**
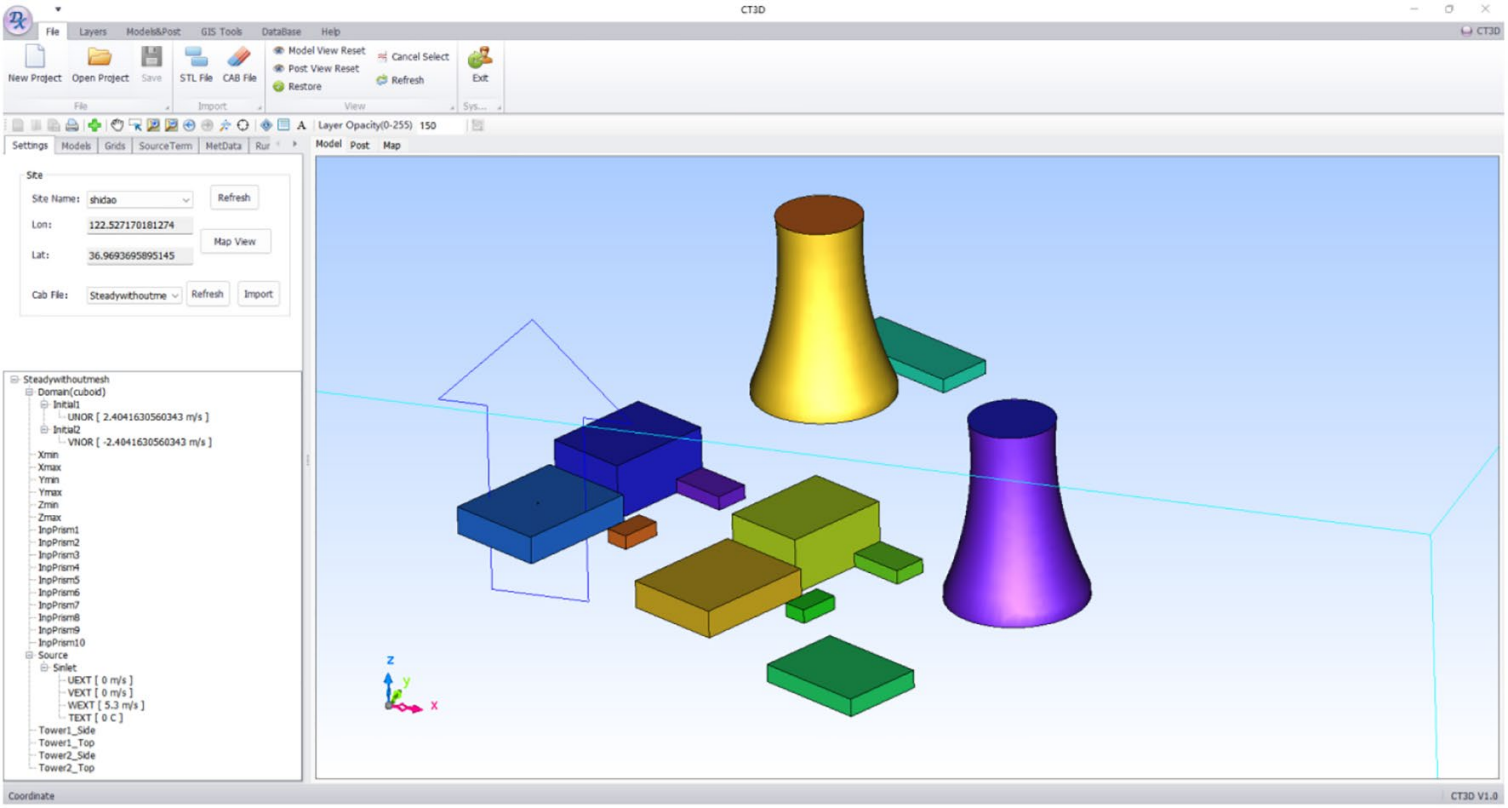
System main interface.

XJCT-3D is developed in VB.net 2010 language, and the user visualization component is Dev Express 14.1.4. The software is designed with the Ribbon style of Office2010, and the main menus include File, Layers, Models & Post, GIS Tools, Database, and Help. Database, Help. We adopt the way of calculation wizard to guide the user to complete the calculation and analysis, all the processes of calculation and analysis only need to input simple parameters to complete a simulation analysis, which greatly enhances the efficiency of calculation. Figure 4 gives the main interface of the system.

There are several specific reasons for choosing VB.net 2010 as the language for developing this software. Firstly, VB.net is known for its simplicity and ease of use, which makes it suitable for both beginner programmers and experienced developers. This language provides a clear and intuitive syntax that allows for faster development and fewer errors.

Another reason for using VB.net is its strong integration with the .NET framework. This framework offers a wide range of pre-built functionalities and libraries that can greatly accelerate development time. Since VB.net is specifically designed to work with the .NET framework, it can seamlessly utilize these resources, resulting in more efficient and robust software.

Moreover, VB.net 2010 supports Rapid Application Development (RAD) features, such as drag-and-drop controls and built-in components. This enables faster prototyping and development cycles, which can be advantageous when time is a critical factor.

Lastly, VB.net provides extensive support for graphical user interface (GUI) development. It allows developers to easily create visually appealing and user-friendly interfaces, enhancing the overall user experience of the software.

The XJCT-3D software stands out from similar software due to several reasons. First and foremost, its interface is highly user-friendly, making it easy for both beginners and experts to navigate and utilize its features effectively. Additionally, XJCT-3D offers a powerful and comprehensive set of tools and functionalities that surpasses what similar software provides.

One of the special advantages of XJCT-3D is its exceptional accuracy and precision in creating 3D models. The software utilizes advanced algorithms and calculations, resulting in highly realistic and detailed representations. This accuracy is crucial for industries like architecture, engineering, and design, where precision is of utmost importance.

Another noteworthy advantage of XJCT-3D is its strong compatibility and integration capabilities. It seamlessly integrates with various industry-standard formats, allowing users to import and export their projects effortlessly. This compatibility ensures a smooth workflow and enhances collaboration between different software and systems.

Furthermore, XJCT-3D offers superior rendering capabilities and a wide range of rendering options. Users can choose from multiple lighting effects, materials, and textures, enabling them to create stunning visualizations of their models. Additionally, the software supports real-time rendering, providing users with immediate visual feedback as they make modifications and adjustments.

Lastly, XJCT-3D excels in providing top-notch customer support and regular updates. The software development team is highly responsive, addressing any technical issues or inquiries promptly. Regular updates with new features and enhancements further enhance the software’s performance and keep it ahead of similar offerings.

### Site selection

Json data^18^ form from the database query to get the latitude and longitude coordinates of the plant site, and then feedback to the system, while the plant site associated cab model data files through python requests library from the server for automated capture, the server access address is defined in the ipconfig.txt file, the key implementation code is as follows.
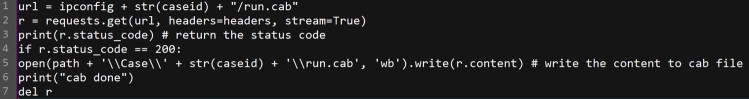


### Models

The large natural draft cooling tower is in typical hyperbolic form, so the 3D model of the cooling tower cannot be obtained directly by using the traditional stretching method. In this system, the modeling process is automated by setting the key parameters of the cooling tower and referencing the python interface provided in free CAD. The key control parameters include the bottom 0 m radius, throat height, outlet radius, and cooling tower height of the cooling tower, and the key implementation code is as follows:
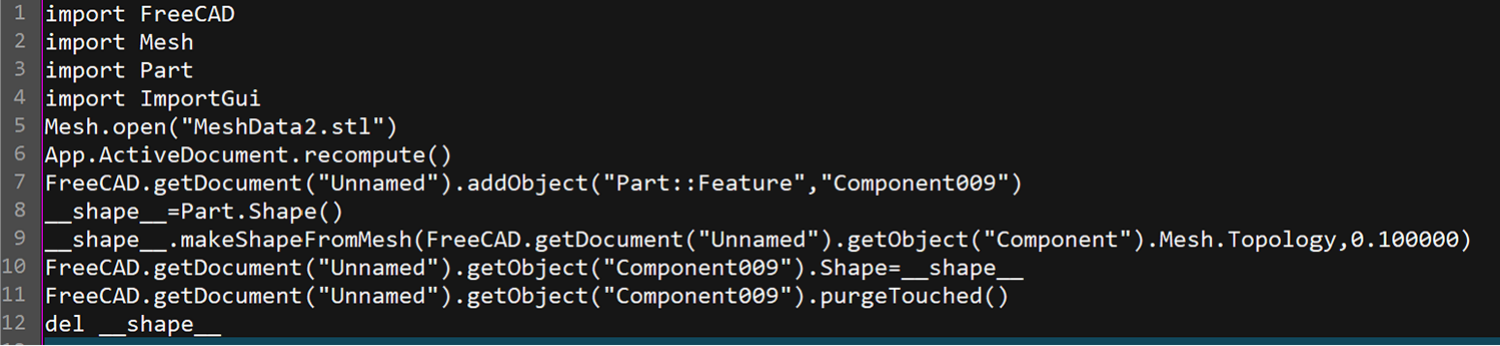


The generated model is converted to x_t format and can be directly imported into XJCT-3D software.

### Mesh

Considering that the atmospheric dispersion of the cooling tower involves a large area, generally more than 10 km, we use a hexahedral mesh in XJCT-3D software to control the number of meshes and improve the calculation efficiency.

Hexahedral mesh was selected due to its advantages in maintaining orthogonality and accuracy in capturing complex geometry when compared to other types of meshes. Furthermore, it has been noted in previous studies that hexahedral meshes can improve computational efficiency and reduce discretization errors in computational fluid dynamic simulations. Therefore, it was deemed to be a suitable choice for the development of the open GIS-based 3D simulation software for predicting cooling tower drift diffusion.

Hexagonal meshes offer a more efficient and structured way to represent complex geometries compared to other mesh types, such as triangular or tetrahedral meshes. The regularity and symmetry of hexagons enable a higher degree of packing efficiency and reduced computational complexity.

For instance, when simulating fluid flow in a porous medium, such as underground reservoirs or oil fields, hexagonal meshes provide a more accurate representation of the complex, interconnected pore spaces. The uniformity of hexagonal cells allows for a closer approximation of the actual pore geometry, resulting in more accurate predictions of fluid behavior and flow dynamics.

Additionally, hexagonal meshes exhibit a higher resolution and connectivity compared to other mesh types. With triangles or tetrahedra, the connectivity between neighboring cells is limited, leading to potential discrepancies when capturing intricate features of the domain. In contrast, hexagonal cells share more boundary faces with adjacent cells, promoting smoother transitions and improved representation of the geometry.

Furthermore, hexagonal meshes offer better numerical diffusion properties compared to other mesh types. Due to the evenly distributed edges and faces, hexagons minimize numerical diffusion effects, which can cause inaccuracies in simulations involving gradients or steep changes in variables.

**Figure 5:**
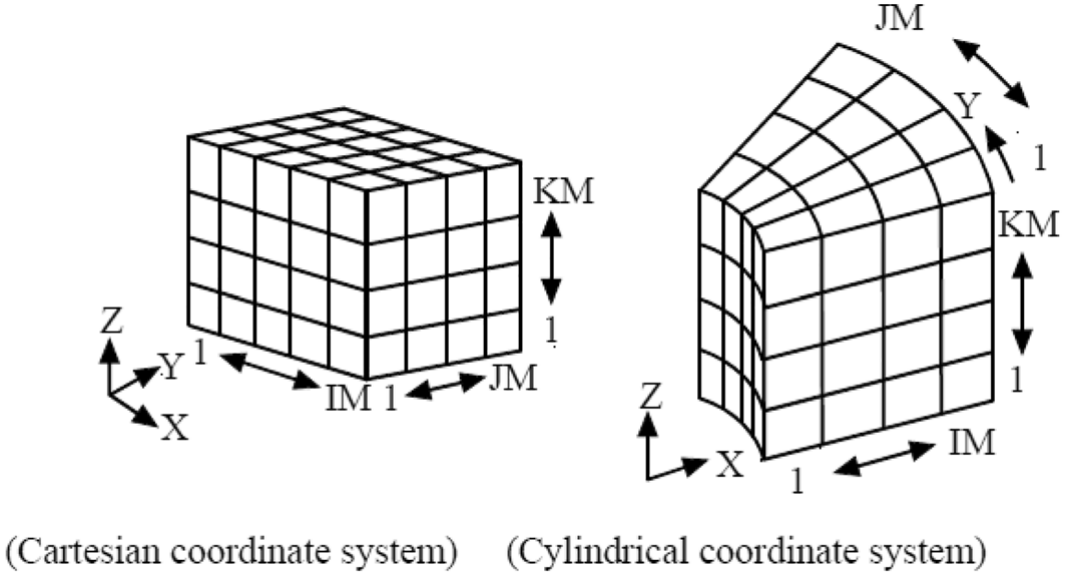
Grid coordinate system.

The analysis region is divided nonuniformly in the respective X, Y, and Z directions as shown in Fig. 5. This division is called mesh division, and the smallest unit to be divided is called mesh element or simply element.

**Figure 6:**
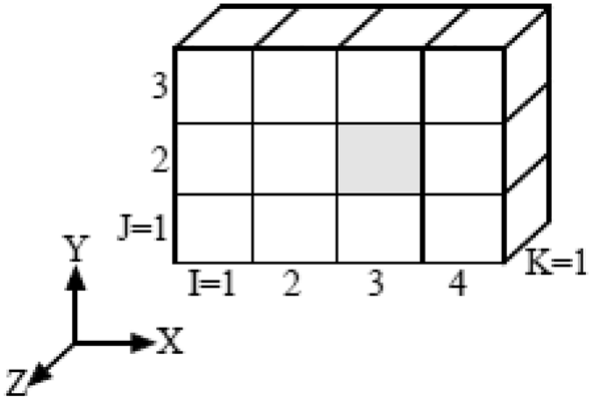
Location of element.

A location of an element can uniquely be determined by the I-th number in the X-direction, the J-th number in the Y-direction, and the K-th number in the Z-direction, so the address of the element can be displayed as (I, J, K), which is called element address. For example, when the element colored in gray in Fig. 6 is located in the address of I = 3, J = 2, and K = 1, the element address is represented by (3, 2, 1).

Each element is surrounded by six faces. Locations of these faces can also be specified similarly. At that time, there are two methods to describe the location of the face.

**Figure 7:**
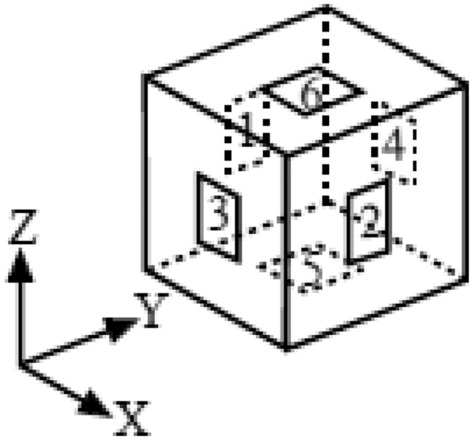
Face number.

The first method is to specify six faces in an element through individual face numbers. As is illustrated in Fig. 7, each face number in the specific element is defined as that face 1 is a face in the negative X-direction, face 2 in the positive X-direction, face 3 in the negative Y-direction, face 4 in the positive Y-direction, face 5 in the negative Z-direction, and face 6 in the positive Z-direction, respectively. These numbers are called face numbers and this method is called specification by face number.

**Figure 8:**
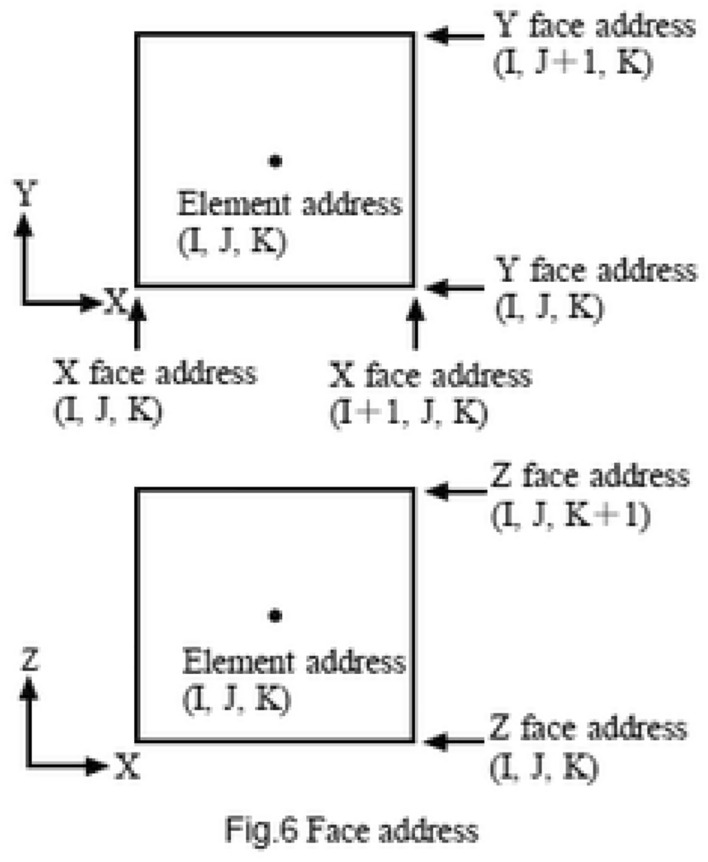
Face address.

The second method is to directly assign address information to faces. Individual elements are surrounded by faces normal to the X-direction (called X face), the Y-direction (called Y face), and the Z-direction (called Z face). Thus, we assign the number 1, 2, and 3 (face direction) to those three directions, specify one of these three numbers, and give a location by using three natural numbers shown in Fig. 8. This is called face address, and this method is called specification by face address.

In the context of XJCT-3D software, the use of a hexagonal mesh provides several benefits in terms of efficiency calculations and accurately capturing phenomena in the dispersion atmospheric problem.

Firstly, the hexagonal mesh division plays a crucial role in improving efficiency calculations. The regular and structured nature of hexagonal cells allows for more effective utilization of computational resources. The evenly distributed cells facilitate a balanced workload distribution, reducing computational time and memory requirements compared to irregular mesh types.

Moreover, the hexagonal mesh division enables more accurate capturing of dispersive atmospheric phenomena. When simulating dispersion, the interaction and movement of pollutants in the air are of primary importance. Hexagonal cells offer a more realistic representation of the dispersion process by closely approximating the continuous nature of air movement.

To ensure that the hexagonal mesh properly captures the phenomena in the dispersion atmospheric problem, several considerations should be taken into account.

Firstly, appropriate refining techniques should be employed to adjust the mesh resolution in regions where strong dispersion or concentration gradients are expected. This refinement ensures that the hexagonal cells are smaller and more concentrated in areas of interest, enabling a better representation of localized phenomena.

Secondly, the choice of appropriate boundary conditions is crucial. The boundaries of the computational domain should be selected in a way that allows for the accurate representation of real-world atmospheric conditions. This could include considering factors such as wind direction, speed, and atmospheric stability.

Lastly, validation and calibration of the hexagonal mesh model against experimental or observational data should be performed. By comparing simulated results with real-world measurements, the accuracy and reliability of the hexagonal mesh representation can be assessed and adjusted if necessary.

The specific meshing parameters are shown in Table 1.

**Table 1:** Meshing parameters.

Field	Value (m)
Calculate the mesh size in the x-direction of the domain	50
Calculate the mesh size in the y-direction of the domain	50
Calculate the mesh size in the z-direction of the domain	50
z-direction grid increment ratio	1.2
Average building surface grid size	1
Grid resolution at release point	0.5

**Figure 9:**
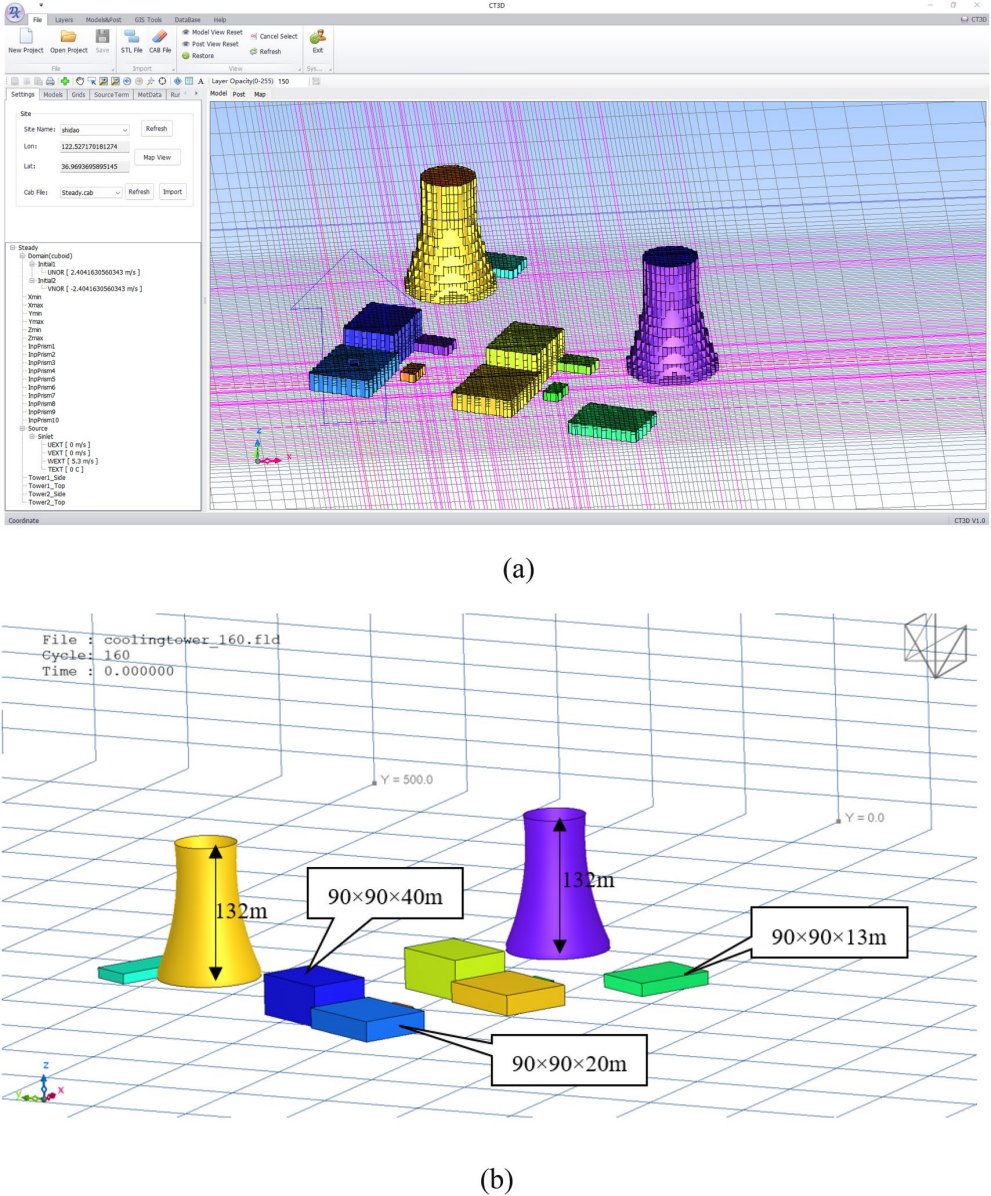
Grid model and model size.

The cooling tower mesh model generated by XJCT-3D software is shown in Fig. 9. It can be seen that the grid model maintains the outline of the original structure better.

### Calculation

**Figure 10:**
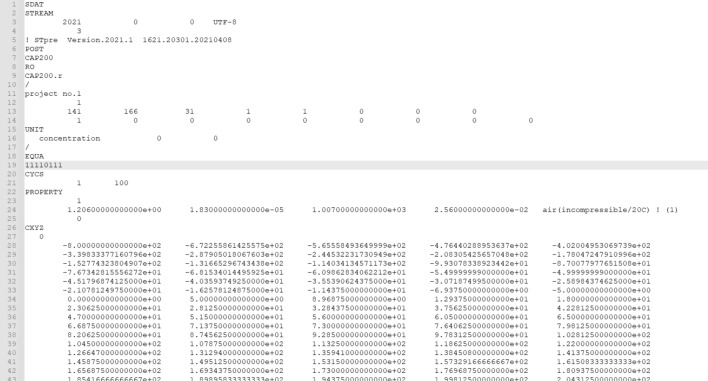
Format of the s file.

The computational control of XJCT-3D software is defined in the .s file as shown in Fig. 10. Through the .s file, the file path of the computational mesh, the record of the computational iteration process, Data type specification, Initial settings, and Command data are recorded.

### Post process

**Figure 11:**
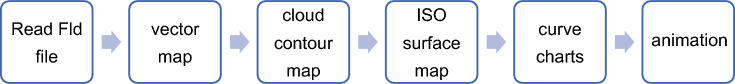
XJCT-3D software post-processing process.

The XJCT-3D software uses the XJ-Post post-processing module to automate the analysis of the calculation results. In this module, we use VB.net development language to realize the generation of wind velocity vector maps, cloud maps, contour surface maps, streamline maps, animations, and axial concentration maps as shown in Fig. 11.

The data processing process is as follows:

1. *Read the fld file* fld is the calculation result file of XJCT-3D software. fld file is saved in binary format, which records the information of the model’s mesh, calculation result data, and so on.
2. *Generate wind field vector map* The wind field vector map is mainly generated using the SetVectorVariable() method provided in Cradle CFD. The plotting area of the wind speed vector is set by the SetPosition() method and the color legend of the vector map is set by the GetObjectColorbarVector() method.
3. *Generate cloud maps* The cloud map of the concentration or wind field is mainly implemented using the CreateObjectSurface() method and the color configuration legend displayed is implemented using the GetObjectColorbarScalar() method. The SetScalarVariable(ScalarName) method also enables the plotting of different pollutant objects.
4. *Generate ISO surface map* ISO surface map is a representation of the isosurface in 3D space, which can give the diffusion range and lifting height of the cooling tower wet plume in 3D space. ISO surface map is drawn by CreateObjectIsosurface() method, and the pollutant to be drawn is set by SetBaseVariable() method. SetBaseVariable() method to set the name of the contaminant to be mapped, and SetBaseValue(curval) method to set the concentration value of the specific isosurface, where curved represents the value of the isosurface. SetMeshFillDisplay(1) method to control the display state of the contoured mesh, set to 1 to display the mesh, set to 0 not to display the mesh.
5. *Generate flow charts* Streamlines can visually track the diffusion trajectory of the cooling tower wet plume. The streamlines can be quickly drawn by the method Fld. represents the X-minimum distance from the computational domain where the streamlines are drawn, and the streamlines show the evolution of the entire wind field in the computational domain.
6. *Generate animation* Animation file by loading the sta configuration file to achieve, through the Fld.ApplySTA(StaFile, False) method can quickly in the software to generate the cooling tower wet plume diffusion process animation, where StaFile represents the sta configuration file path, False represents not in the animation file to show the path of the fld file.

### GIS couple

XJCT-3D software uses the open source Dotspatial^19^ component library, DotSpatial is a core GIS library based on the NET 4.0 framework platform, which brings together various. NET-based GIS components in one, such as SharpMap, and Proj. DotS partial is an upgraded version of MapWindow^20^. The functionality is more complete than MapWindow, and the component can be added to the development language platform. NET language compatible language platforms, such as Visual Basic.NET, Visual C#, NET, etc. The included geographic information data processing components are suitable. The core component library of DotSpatial is DotSpatial.Controls.dll, which is similar in functionality to Mapinfo’s MapX component^21^ and integrates most of the MapWindow GIS features, such as attribute table editing and shapefile editing. The component library supports a variety of GIS map formats, including shape files, Geotiff, ESRI Arcinfo ASCII and binary grids^22^.

1. *Loading of class libraries* DotSpatial is a foreign open source software, the core of its components is DotSpatial.Controls.dll dynamic link library. It features a completely open code, users can download the source code from its official website for personal modification and research, and development, the download address is https://DotSpatial.codeplex.com/. If you make sure that DotSpatial has been downloaded on your machine, select New to add DotSpatial.Controls.dll in the VS2010 toolbox. Controls.dll contains a series of basic controls that we need for GIS desktop development, such as MAP, Legend, etc. We can simply drag and drop to use them.
2. *CFD results conversion* Since the results generated by CFD software are binary fld format files, which cannot be loaded by the DotSpatial component library, the data need to be converted to display properly. GetVariableInfo method provided by Cradle CFD to convert the data, extract the pollutant concentration result data to csv format data file for a certain plane, and then use the map interface of DotSpatial to realize the conversion and loading of the data in GIS, the core code is as follows:

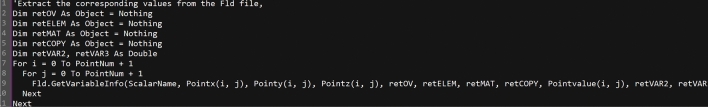

(3) *Harmonization of map formats* Since DotSpatial only supports ERSI’s Shp format maps, the preparation work before system development is to make Shp format maps, such as dwg format and Tab format can only be converted to Shp format first. Dwg maps should first use MapInfo’s powerful mapping features to add the attribute information we need, save it in TAB format, then convert it to the intermediate data format MIF, and then use MapInfo’s universal conversion tools to convert it to the ERSI standard Shp format. Inevitably, there will be data loss in this process. Since the Shp format file cannot save layer color information, all the original color information will be lost after conversion, and we should reset the map color to get a better visual effect. The core code of its implementation part is as follows:

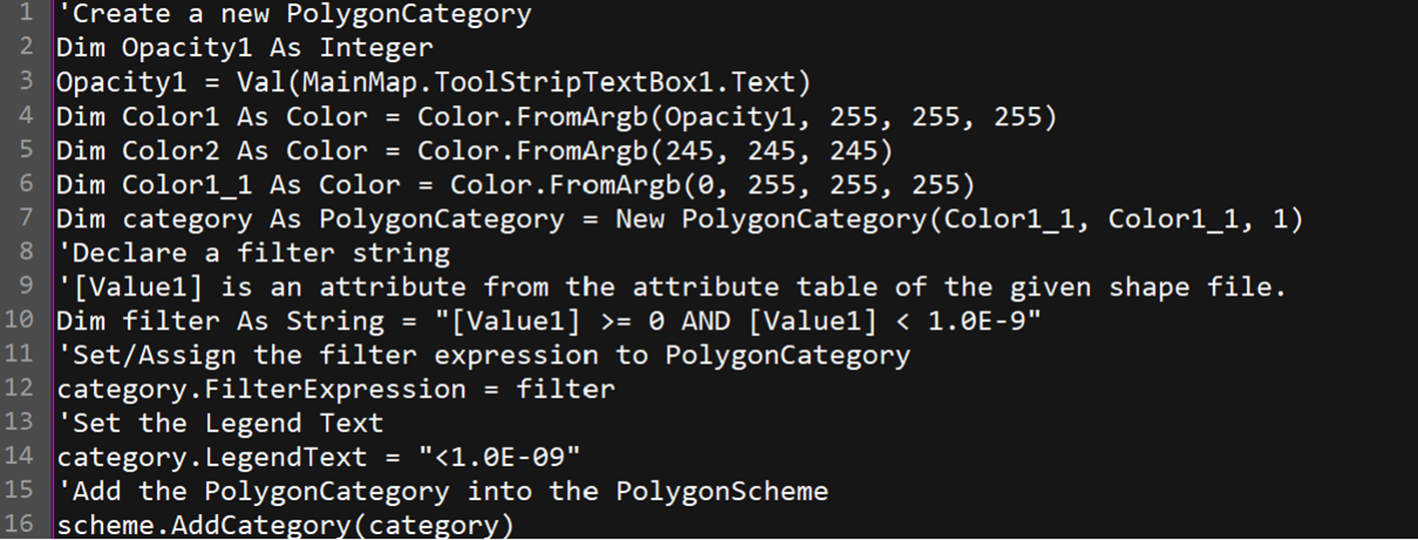

(4) Shp format file will give each category a color, here I assign each category a different single value, then DotSpatial will automatically set a color for each category, to achieve the effect of resetting the color.

### Initial parameters

The initial parameters of XJCT-3D software are as follows:

- Latitude and longitude coordinates of the plant site: 122.5° E, 36.58° N.
- Resolution of the grid model: length 1 m, width 1 m, height 1 m, vertical growth rate 1.2.
- Calculation model: steady state, K-ε turbulence model.
- Number of calculation steps: 100.
- Output results: wind field, concentration field, ISO Surface

## Case study

In this paper, we discuss the results of XJCT-3D software calculations on the example of a small nuclear power reactor site, in which we simulate the scenario of simultaneous release of two cooling towers and verify the validity of XJCT-3D software calculations based on the observed data from tracer experiments.

### Data entry

In the calculation of this case, the wind speed of 10 m height is 3 m/s, which is the annual average wind speed of the plant site area, the wind direction is NE direction, the atmospheric stability is taken as D class stability, and the wind profile index of the incoming flow is taken as 0.25.

“D class stability” refers to a classification system used to assess the stability of the atmosphere. It categorizes atmospheric conditions based on the vertical temperature gradient and the resulting vertical motion of air parcels. D class stability is characterized by a stable atmosphere, where air parcels tend to resist vertical motion, resulting in limited turbulence and smooth airflow^12^.

On the other hand, the ‘wind profile index’ is a measure used to evaluate the vertical variation of wind speed with height. It provides insights into the characteristics of the wind profile within a specific area. The index takes into account factors such as wind shear, veering, and magnitude, which can influence the behavior and impact of wind flows in different scenarios.

According to the results of on-site turbulence observation, the turbulence length at the entrance of the whole calculation domain is 50 m, and the turbulence intensity is taken as 0.1. The turbulence length at the exit of the cooling tower is taken as 7 m, and the turbulence intensity is taken as 0.1. The land use type in the area around the plant site is mainly farmland and forest land, so its surface roughness is taken as 0.3 m. Considering the steady-state release, the emission rate of the cooling tower heat plume is 5.3 m/s.

It is assumed that the flow on the outlet surface is fully developed (normally 5 times distance of cooling tower height) and the flow has returned to normal flow without building obstruction, so the relative pressure at its outlet boundary is zero and the building surface is a smooth wall with friction. The assumption of fully developed flow is a common simplification used in fluid dynamics and is based on the assumption that the flow has had sufficient distance to approach a steady-state condition. In the case of cooling towers, the outlet surface is typically far enough from the cooling tower fill and other components that the flow can be considered fully developed. Additionally, the fully developed flow assumption is also supported by previous studies that have investigated the flow characteristics in cooling towers.

Although the fully developed flow assumption is a simplification, it has been shown to provide reasonable accuracy in many engineering applications, including cooling tower simulations. However, we acknowledge that there may be cases where the fully developed flow assumption may not be valid, such as in situations where there are significant flow disturbances or non-uniform outlet surfaces. In such cases, it may be necessary to use more complex flow models to accurately capture the flow behavior.

### Computational simulation

**Figure 12:**
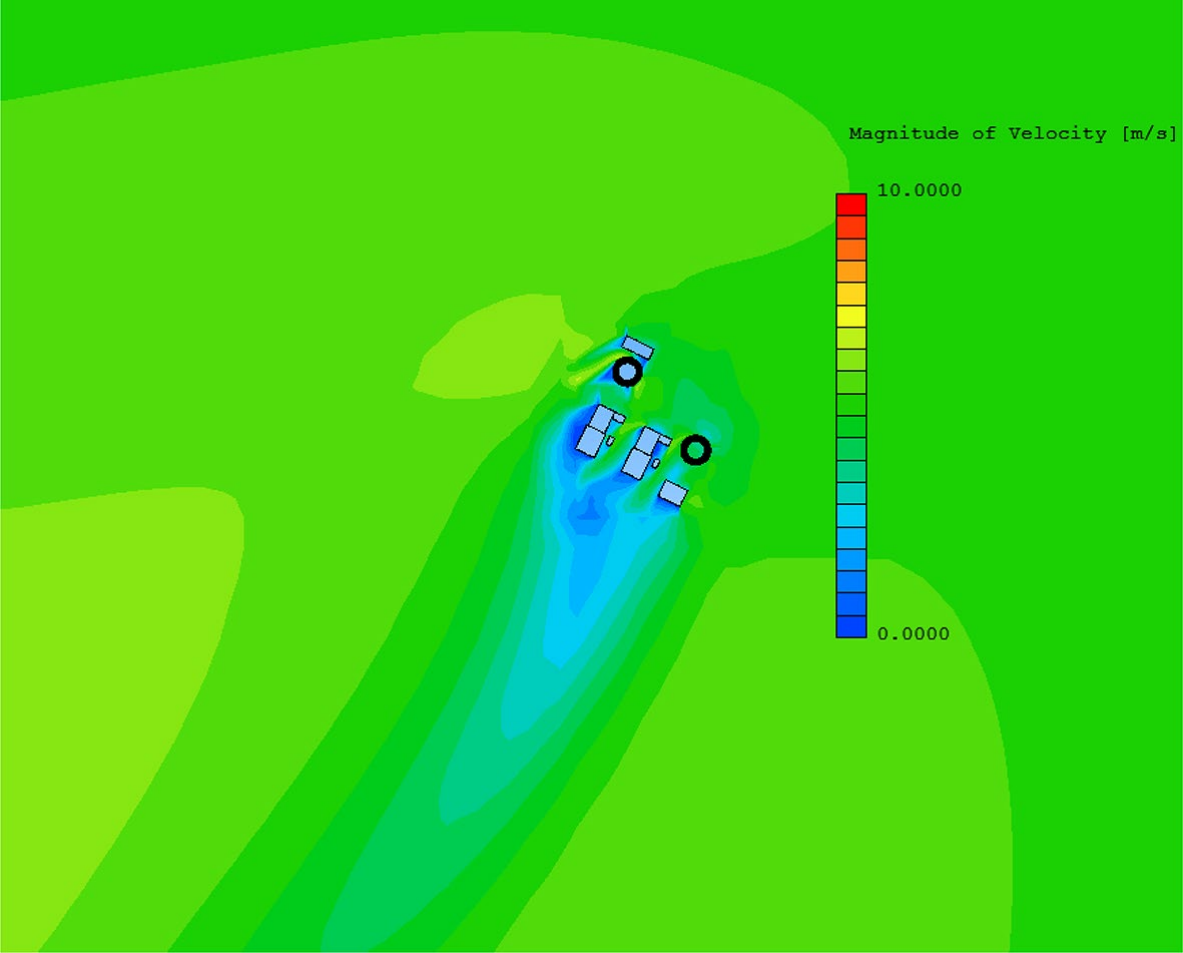
Wind field display.

When all the data are prepared, click the Run button in XJCT-3D software to start the cooling tower thermal plume simulation and analysis calculation. Figure 12 gives the results of the calculated wind field. It can be seen that an accelerated airflow is formed on both sides of the cooling tower, and in the leeward area of other structures in the plant, a cavity area of different degrees is formed, and the wind speed in the cavity area becomes smaller than the incoming flow.

**Figure 13:**
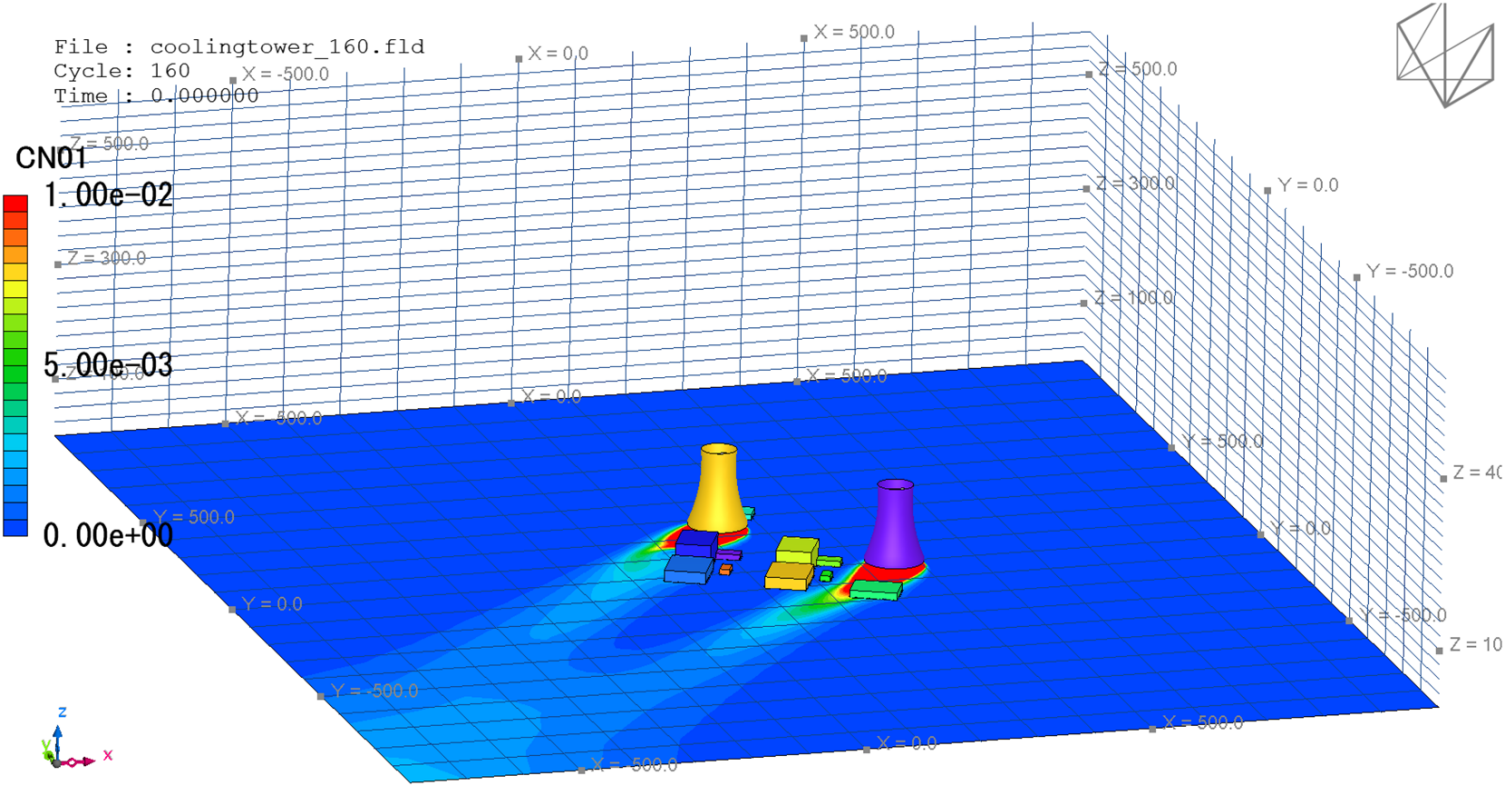
Concentration cloud map.

Click the Contour-Pollu button in the Plot ribbon to generate a distribution cloud of the cooling tower heat plume, as shown in Fig. 13.

**Figure 14:**
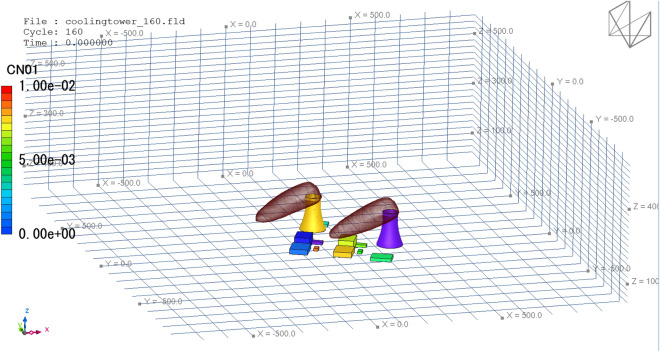
3D animation.

Click the Load button in the Animation File ribbon to generate a flow animation and a diffusion animation of the entire computational domain, as shown in Fig. 14.

**Figure 15:**
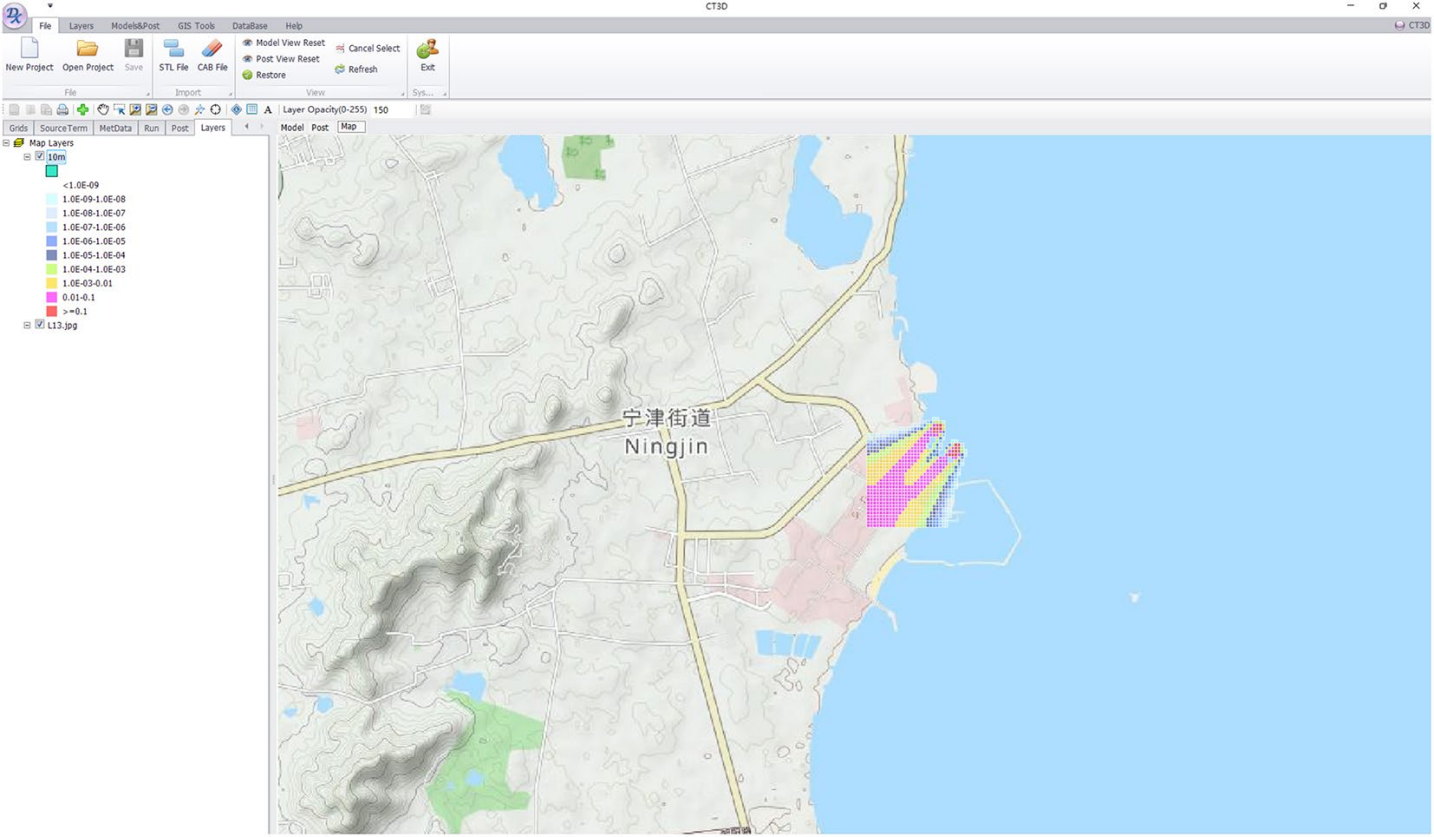
GIS coupling analysis.

Click the Open in Map button to set the area and height of the imported layer to be imported into the GIS map, click the Output button, XJCT-3D software automatically extracts the data of the CFD corresponding height layer and converts it to shp format for loading and displaying, as shown in Fig. 15. In the Map Layers component of the map display area, you can also customize the display color of the map, and export the map file for loading into third-party software, such as SuperMap and Mapbox, for re-analysis.

### Comparison analysis

**Figure 16:**
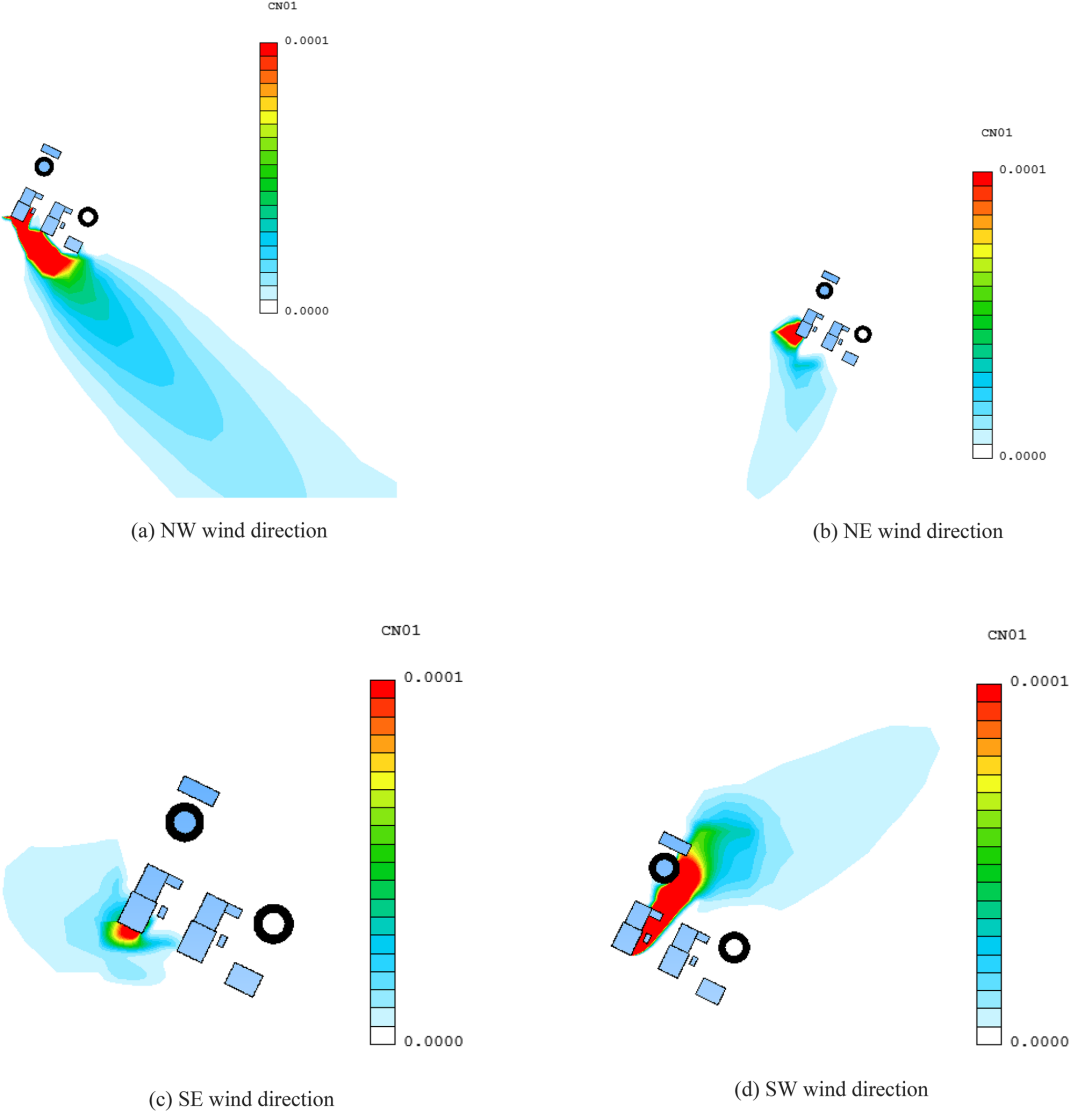
Concentration field distribution at the height of 10 m.

Figure 16 shows the distribution of pollutants at the height of 10 m under different wind directions. It can be seen that due to the influence of the structure of the plant, the diffusion of pollutants in the downwind direction has a certain deflection. The deflection of NE wind direction and SE wind direction is the most obvious. The main reason is that in the two wind directions, a certain area of deflection airflow is formed between Unit 1 and Unit 2, which causes the diffusion of pollutants to deflect.

**Figure 17:**
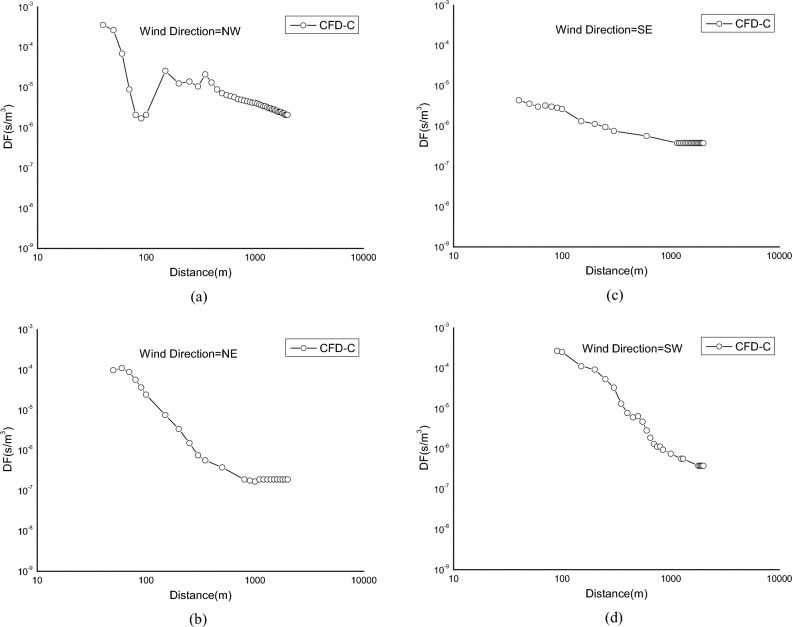
Axis concentration curve.

Figure 17 shows the distribution of the atmospheric dispersion factor of the downwind axis under four wind direction conditions. It can be seen that for the NW wind direction, the maximum concentration point appears at a wind direction of 40 m below the release point, which is 3.55E−04 s/m^3^. For the NE wind direction, the maximum concentration point appears at 60 m below the release point, which is 1.11E−04 s/m^3^. For the SE wind direction, the maximum concentration point appears at 40m below the release point, which is 4.34E−06 s/m^3^. For SW wind direction, the maximum concentration point appears at 90 m downwind from the release point, which is 2.70E−04 s/m^3^. At the same time, it can be seen that for the NE and SW wind directions, the concentration of pollutants decreases rapidly with the increase of the downwind distance. For the SE wind direction, the concentration of the ground pollutants decreases not so obviously with the downwind distance, this is mainly related to the relative position of the site structure and the release point. In the NE wind direction, due to the cooling tower blockage of the upstream, the airflow at the release point becomes weak, the high concentration contaminated area is formed at the near zone, and for the SW wind direction, due to the blockage of the steam turbine plant, the pollutants are retained in the near area, thus forming a high concentration area. For the SE wind direction, the cooling tower has less impact on the diffusion of pollutants, and more is the downwashing effect of the reactor building, so the concentration of pollutants reaching the ground is low. Under the condition of NW wind direction, the concentration distribution of the pollutants in the downwind direction firstly decreased, then increased and then decreased. The main reason is that the pollutants are unevenly distributed due to the influence of the disturbed airflow between units 1 and 2.

## Results and discussion

The XJCT-3D software implements a three-dimensional simulation analysis of the cooling tower thermal plume with the following features:

- The integrated modeling, meshing, computation and analysis, and post-processing processes are realized, which significantly improves the efficiency of conducting simulation calculations and reduces the difficulty for users to carry out computation and analysis.
- Support seamless integration of CFD calculation results with GIS geographic information, which makes it easier for users to visualize and analyze the data.
- The optimized grid algorithm model is used to significantly improve the overall computational analysis efficiency. The face number is defined as the number of surfaces in the cooling tower that the air stream contacts. A higher face number typically leads to better thermal performance, as it increases the heat transfer area and reduces the air velocity at each individual surface. Additionally, higher face numbers also reduce the overall drift loss of the cooling tower. Our simulation software considers the face number as an important parameter, and we have conducted simulations using different face numbers to evaluate the cooling tower performance. In our research, increasing the face number leads to a decrease in drift loss and an overall improvement in the cooling tower’s performance. For example, for a single natural ventilation cooling tower, when the grid is 1.2 million, the calculation accuracy is 1.7 times higher than that of a 600,000 grid, but when the grid is increased to 2.4 million, the calculation accuracy is only 0.02 times higher, indicating that for a single natural ventilation cooling tower, a grid of 1.2 million is an ideal value.

The validity of the calculations of the XJCT-3D software was verified using experimental observations from the ChalkPoint power plant^10^, In the ChalkPoint experiment, wet deposition was monitored at downwind distances of 0.5 km and 1.0 km, below is a comparison of XJCT-3D result and ChalkPoint experiment data.

**Figure 18:**
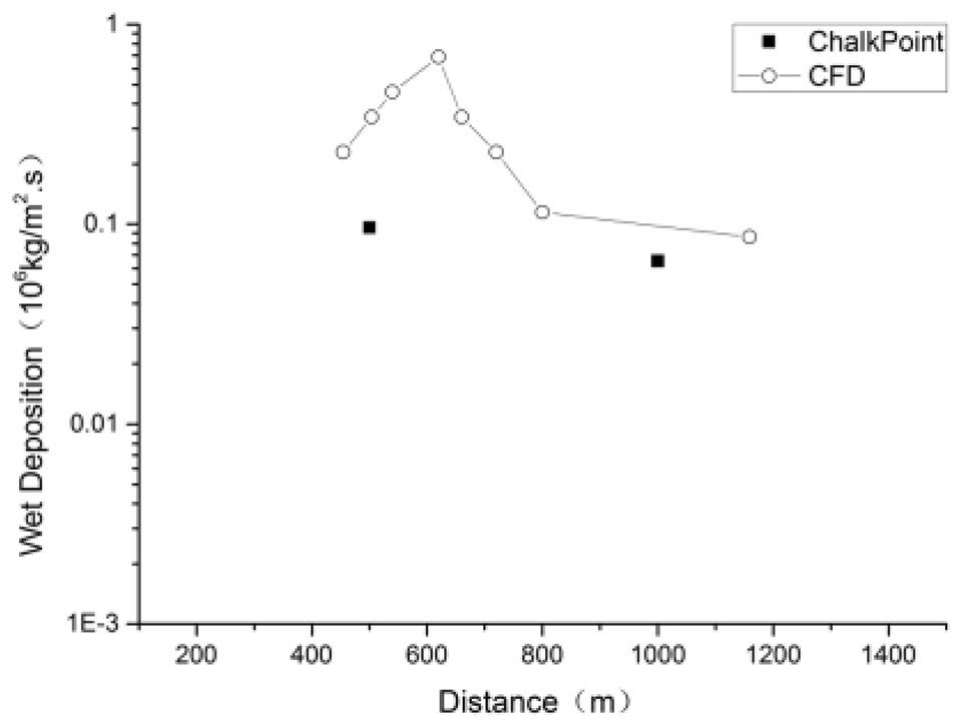
Wet deposition data comparison.

As can be seen from Fig. 18, the results obtained from XJCT-3D calculations are in good agreement with the results of ChalkPoint tracer experiments, and the deviation of the whole model is within 50%. Also from the XJCT-3D calculation results, it can be seen that the maximum value of the cooling tower thermal plume wet deposition occurs near 610 m with a maximum value of 6.9E−07 kg/m^2^ s. The results of the computational validation of XJCT-3D are in good agreement with those of Meroney^12^ and are indeed slightly higher than observation data.

In the study conducted using the self-developed XJCT-3D software, which is based on Cradle CFD, the main objective was to simulate and predict the diffusion of cooling tower drift in a three-dimensional (3D) space. The cooling tower thermal plume is the upward rising column of warm air and moisture emitted from the cooling tower. Wet deposition refers to the process of particles or droplets in the plume settling onto surfaces, including the ground, buildings, or nearby water bodies.

The observed maximum value of cooling tower thermal plume wet deposition at 610 m indicates the highest concentration or deposition rate of particles or droplets at that specific height in the simulated environment. This finding suggests that the cooling tower emissions carry a significant load of particles or droplets that have settled on surfaces at this particular altitude.

The understanding of the maximum value of wet deposition at 610 m is crucial as it provides insights into potential environmental and human health impacts. It helps in identifying and assessing areas at relatively higher risk of deposition, such as nearby ecosystems, farmland, or urban areas. This information can contribute to the development of effective mitigation strategies and the implementation of appropriate measures to minimize the impact of cooling tower emissions.

Comparing the XJCT-3D model developed in this paper with the Fluent computational model used by Meroney^12^, the main improvements include the following:

1. The computational efficiency of the model. The computational efficiency of XJCT-3D is more than two times faster than Fluent for the same number of meshes.
2. XJCT-3D takes 1 s to divide 1 million grids, while Fluent takes at least 2 min to do so.
3. GIS data integration display, XJCT-3D’s calculation results can be directly displayed in the GIS platform, while Fluent does not have such a function.

XJCT-3D, the software developed for this research, offers superior computational efficiency for several reasons:

1. *Parallel computing* XJCT-3D utilizes parallel computing techniques to distribute computational tasks across multiple processors or cores. This enables the software to harness the power of parallel processing and significantly reduce the computational time required for simulations. Compared to traditional serial computing, parallel computing allows for faster and more efficient calculations, especially for complex and computationally intensive simulations.
2. *Grid optimization* XJCT-3D incorporates algorithms that optimize the computational grid used in the simulations. By adapting the grid resolution to the specific needs of the problem, the software achieves a balance between accuracy and computational efficiency. It dynamically adjusts the grid resolution based on the flow characteristics and ensures that the computational resources are efficiently allocated where they are most needed.
3. *Solver efficiency* The software employs advanced solver algorithms that are highly efficient in solving the fluid flow equations. These solvers are designed to handle complex geometries, turbulent flows, and multiphase phenomena, which are typical in cooling tower drift diffusion simulations. The algorithms are optimized to achieve fast convergence and reduce the computational effort required to obtain accurate results.
4. *Optimized data processing* XJCT-3D utilizes optimized data processing techniques to efficiently handle and process large volumes of simulation data. This includes data input/output operations, post-processing tasks, and storage management. By minimizing the data processing time, the software further enhances its computational efficiency.

## Conclusion

The computational analysis of large naturally ventilated cooling tower plume dispersion plays a crucial role in assessing the local environmental impact of the shadow effect and deposition processes that occur during cooling tower operation. The CFD fluid dynamics approach is a reliable computational evaluation model for conducting cooling tower plume dispersion analysis. The key contribution of this paper lies in the development of the XJCT-3D simulation and analysis software for integrated cooling tower plume dispersion simulation. This software is based on the Cradle CFD computational engine and is coupled with the Open GIS component Dotspatial, which enables geospatial visual representation of the computational results.

XJCT-3D software can realize the rapid construction of a three-dimensional structure model, the rapid division of the grid, the calculation efficiency is greatly improved, and the calculation results can be drawn with one click to generate a wind field vector map, pollutant distribution cloud map and the dynamic diffusion display of cooling tower wet plume.

FreeCAD is driven by the Python language to automate the construction of the cooling tower 3D model, based on the VBS API interface provided in Cradle CFD. This enables fast simulation calculations and automated post-processing analysis in XJCT-3D software. Based on the API interface provided by Dotspatial. The CFD calculation results can be displayed directly in the GIS platform.

The validity of the XJCT-3D software simulation was verified using tracer experimental data from the ChalkPoint power plant, and the conclusion showed that XJCT-3D accurately models wet plume deposition during cooling tower operation. Also from the XJCT-3D calculation results, it can be seen that the maximum value of the cooling tower thermal plume wet deposition occurs near 610 m with a maximum value of 6.9E-07 kg/m^2^ s.

The development of the XJCT-3D software in the context of this research paper is significant for several reasons:

- *Unique capability* XJCT-3D introduces a unique capability to perform 3D simulations in the field of cooling tower drift diffusion. This software fills a gap in the existing tools available for this specific application, allowing researchers and practitioners to conduct more accurate and detailed simulations of cooling tower drift diffusion phenomenon. The software’s specialized features and functionalities make it a valuable addition to the existing scientific toolbox.
- *Improved accuracy* XJCT-3D incorporates advanced computational fluid dynamics (CFD) techniques and algorithms tailored specifically for cooling tower drift diffusion simulations. By utilizing these state-of-the-art methods, the software enhances the accuracy of predictions and provides researchers with more reliable results. This improvement in accuracy enables better understanding and analysis of the cooling tower performance, leading to improved design and operation strategies.
- *Enhanced efficiency* The XJCT-3D software offers computational efficiency advantages, as detailed earlier in my previous response. Its ability to efficiently handle complex geometries, turbulent flows, and multiphase phenomena ensures that accurate simulations can be conducted within reasonable timeframes. This efficiency allows researchers and engineers to explore different scenarios, optimize designs, and make informed decisions more effectively.
- *Open GIS integration* XJCT-3D’s integration with open Geographic Information Systems (GIS) platforms enhances its usefulness and accessibility. This integration enables the utilization of real-world geographical and environmental data in the simulations, providing a more realistic context for analysis. The ability to incorporate GIS data into the simulations allows researchers to study cooling tower drift diffusion in specific locations, considering factors such as wind patterns and land use, leading to more accurate predictions and informed decision-making.

In the subsequent research, we hope to continuously update the XJCT-3D software, to achieve the following objectives.

1. *Objective 1* Enhance the modeling techniques
  Investigate and implement advanced computational fluid dynamics (CFD) techniques specific to cooling tower drift diffusion simulations.
  Explore strategies to improve the accuracy and efficiency of the software's modeling capabilities.
  Incorporate additional multiphase phenomena modeling features into the software to capture more realistic cooling tower behavior.
2. *Objective 2* Validate and verify the software.
  Conduct validation studies by comparing XJCT-3D simulated results with experimental data and/or benchmark simulations.
  Perform verification analyses to ensure the software produces consistent and reliable results.
  Establish a comprehensive validation framework to assess the accuracy and robustness of the software across different scenarios.
3. *Objective 3* Expand application capabilities.
  Investigate the feasibility of applying XJCT-3D to study different types of cooling towers (e.g., natural draft, mechanical draft).
  Explore the software’s potential for analyzing the impact of cooling tower drift diffusion on nearby environments, such as water bodies or urban areas.
  Assess the possibility of integrating XJCT-3D with other software tools or platforms to enhance its functionality and broaden its applications.
4. *Objective 4* Optimize software performance
  Conduct scalability studies to evaluate the software's performance for larger and more complex cooling tower geometries.
  Improve the software's parallel computing capabilities for faster and more efficient simulations.
  Investigate ways to streamline the user interface and workflow, making the software more user-friendly and accessible to a broader range of users.

## Data Availability

The datasets generated during and/or analysed during the current study are available from the corresponding author on reasonable request. Name of software: XJCT-3D; Developer: Dr.Xuan Wang; Contact Address: No.28 Xianning West Road, Xi’an, Shaanxi Province; E-mail address: dazzle@126.com; Date of first release: July 2020; Software required: Cradle CFD 2021, MySQL5.0; Program Language: VB.net; Availability and cost: XJCT-3D is free and can be downloaded from research gate at: https://www.researchgate.net/publication/363622772_XJCT-3D.

## List of symbols

*z*: The lifting length of the visible wet plume in meters (m).
*l*: Length of the visible wet plume in meters (m).
*q*_*p*__0_: Specific humidity of the cooling tower outlet mist plume in grams per gram (g/g).
*q*_*l*__0_: Initial specific humidity of the cooling tower outlet wet plume liquid water in grams per gram (g/g).
*q*_*s*_: Ambient atmospheric saturation specific humidity in grams per gram (g/g).
*q*_*e*_: Ambient atmospheric specific humidity in grams per gram (g/g).
*W*_0_: Initial velocity of the wet plume at the exit of the cooling tower in meters per second (m/s).
*F*_0_: The initial heat flux of the wet plume at the exit of the cooling tower, in meters to the fourth power per cubic second (m^4^/s^3^).
*ρ*: The density of the fluid and is a constant (g/m^3^).
*v*_*x*_, *v*_*y*_, and *v*_*z*_: The velocity vector components of the fluid in the *x*, *y*, and *z* directions, respectively (m/s).
*t*: The time (s).
*τ*_*x*_, *τ*_*y*_ and *τ*_*z*_: The molecular viscous stress components of the fluid in the* x*, *y*, and *z* directions, respectively (m^2^/s).
*λ*: The fluid thermal conductivity term (w/m K).
*S*_*T*_: The heat source correction term of the fluid (w).
*c*_*S*_: The volume concentration of component *S* (l/m^3^).
*ρc*_*S*_: The mass concentration of component *S* (g/m^3^).
*D*_*S*_: The dispersion coefficient of component *S*.
*S*_*S*_: The mass production rate of the component, which is the release rate by atmospheric dispersion (g/s)
